# Etiology of the fourth heart sound is a novel anatomic and pathophysiologic discovery of the heart

**DOI:** 10.1111/anec.13014

**Published:** 2022-10-25

**Authors:** Pekka J. Kuusela

**Affiliations:** ^1^ Private Internist Kuopio Finland

Dear Editor,

I read with interest the report of C Sakai and the colleges concerning the fourth heart sound (FHS) and hypertrophic cardiomyopathy (HCM; Sakai et al., 2022). In the formation of the fetal muscular part of the interventricular septum (IVS), the expanding ventricles grow and their medial walls approach and fuse, forming the septum. The inside corner between the septum and the right anterior ventricular wall exhibits the deep pits being called interventricular sinuses (ISs). The IS passes through the right IVS formed from the medial wall of the expanding fetal right ventricle (RV). The opening of the interventricular vein (IV) (kuuselian vessel) is located in the IS between the medial walls of the expanding fetal RV and fetal left ventricle (LV). The IV is not a canal or channel or blood vessel, but a slit between the fibers of the muscle leading to the outer layer of the left central muscular part of the IVS and runs into the LV at an angle of about 90° through the left IVS surrounded by the interventricular sphincter (ISP). The IV exhibits 2–3 oval 2 × 5 mm openings in the left central muscular part of the IVS. Hypoxia may be the physiological factor that recruits IV of the fetal heart and augments the flow of the oxygenated blood from right to left. The ISP and the IV may become patent by relaxing and widening of the helical heart at the right atrial filling phase at the end of the fetal diastole. The sinoatrial node initially activates the right atrium, followed by activation of the left atrium. Left‐to‐right communication does not result as the earliest left ventricular action closes the ISP. Hypoxia may recruit the IV of the heart at the right atrial filling phase and create the venous flow from right to left into the LV. This flow generates the abnormal FHS common in hypertrophy of systemic hypertension and in ischemic heart disease. Noise of the FHS may disturb CW‐doppler measurement (Kuusela, 2014, 2018). High heart rate may also cause negative left ventricular pressure and sucking effect at the early diastole of the normal heart (Udelson et al., 1990). The FHS appears at the 80% submaximal heart rate during a treadmill stress test in normal hearts (Aronow et al., 1971). I do not yet fully understand the anatomy, function, and embryology of the human heart and circulation. The case report (Kuusela, 2018) suggests an unusually plentiful venous flow at the right atrial filling phase from right to left through the IV. Venous blood may reach the brain and the coronary arteries during every cardiac cycle. I do not fully know recruitment of the fetal and adult IV due to fetal physiological and adult pathophysiological reasons. I do not know whether end‐stage HCM exhibits excessive hypertrophy of the ISP and arrhythmogenic septal fascicle of the left His bundle.

## AUTHOR CONTRIBUTION

The article was contributed only by the author.

## CONFLICT OF INTEREST

None.

## ETHICAL APPROVAL

No Patient case was presented by the text (Letter).

## Data Availability

Data opently available in public repository that issues datasets with DOIs.

## References

[anec13014-bib-0001] Aronow, W. S. , Papageorge's, N. P. , Uyeyama, R. R. , & Cassidy, J. (1971). Maximal treadmill stress test correlates with postexercise phonocardiogram in normal subjects. Circulation, 43, 884–888. 10.1161/01.CIR.44.3.397 5578861

[anec13014-bib-0002] Kuusela, P. J. (2014). The heart exhibits right to left communication between the fibres of the muscular part of the interventricular septum. Folia Morphologica, 73(1), 42–50. 10.5603/FM.2014.0006 24590522

[anec13014-bib-0003] Kuusela, P. J. (2018). Interventricular vessel of the heart. Cardiology Research, 9(2), 111–115. 10.14740/cr533w 29755629 PMC5942241

[anec13014-bib-0004] Sakai, C. , Kawasaki, T. , Kawamata, H. , Harimoto, K. , Shiraishi, H. , & Matoba, S. (2022). Absent fourth heart sound as a marker of adverse events in hypertrophic cardiomyopathy with sinus rhythm. Annals of Noninvasive Electrocardiology, 27(3), e12932. 10.1111/anec.12932 35146850 PMC9107087

[anec13014-bib-0005] Udelson, J. E. , Bacharach, S. L. , Cannon, R. O. , & Bonow, R. O. (1990). Minimum left ventricular pressure during beta‐ adrenergic stimulation in human subjects. Evidence for elastic recoil and diastolic “suction” in the normal heart. Circulation, 82, 1174–1182. 10.1161/01.CIR.82.4.1174 1976048

